# Omalizumab in Food Allergy in Children: Current Evidence and Future Perspectives

**DOI:** 10.3390/life15050681

**Published:** 2025-04-22

**Authors:** Cristiana Indolfi, Alessandra Perrotta, Giulio Dinardo, Angela Klain, Carolina Grella, Paola Palumbo, Michele Miraglia del Giudice

**Affiliations:** Department of Woman, Child and General and Specialized Surgery, University of Campania ‘Luigi Vanvitelli’, 80138 Naples, Italy; cristianaind@hotmail.com (C.I.); alessandraperrotta96@gmail.com (A.P.); caro.grella94@gmail.com (C.G.); paolapalumbo1596@libero.it (P.P.); michele.miragliadelgiudice@unicampania.it (M.M.d.G.)

**Keywords:** omalizumab, food allergy, food allergies, food hypersensitivity, immunotherapy, immunoglobulin E, monoclonal antibodies, child, adolescent, quality of life

## Abstract

Omalizumab (OMA) is gaining recognition as a promising therapeutic approach for IgE-mediated food allergies in pediatric patients. We conducted a review analyzing 22 studies, including randomized controlled trials, observational studies, and case reports, to evaluate the efficacy and safety of OMA in food allergy management in children and adolescents. The results indicate that OMA, whether used as monotherapy or in combination with oral immunotherapy (OIT), significantly increases allergen tolerance, reduces the severity of allergic reactions, and improves patients’ quality of life. When used alongside OIT, OMA reduced adverse reactions during dose escalation and maintenance phases, facilitating safer and more effective desensitization. Additionally, OMA demonstrated benefits beyond food allergy management, including improved asthma control and a reduction in food allergy-related anxiety. However, challenges remain, including high costs, the need for standardized treatment protocols, and limitations related to total IgE thresholds for eligibility. While OMA has been FDA-approved for food allergy treatment in the United States, further research is needed to establish long-term efficacy, optimal dosing strategies, and its role in sustained tolerance development. Future research should focus on optimizing treatment protocols and identifying which patients will benefit the most. Integrating omalizumab into food allergy management could revolutionize pediatric care, offering hope for a safer, more effective approach to desensitization.

## 1. Introduction

**Figure 1:**
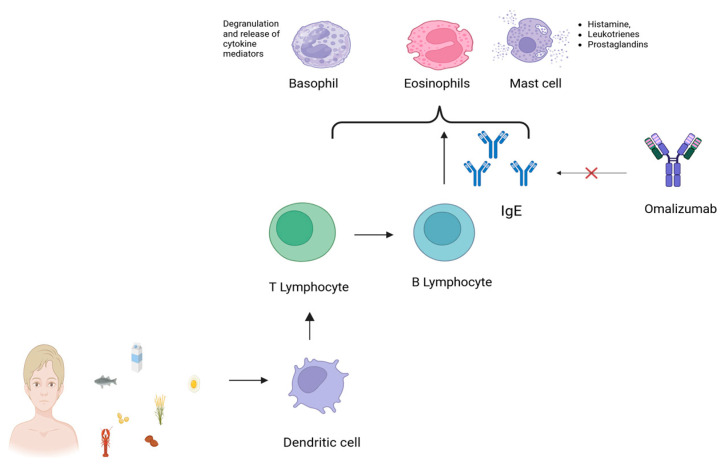
Mechanisms through which Omalizumab acts in reducing food allergic responses.

IgE-mediated food allergies (FAs) affect 5–10% of children in developed countries [1], with manifestations ranging from mild urticaria to life-threatening anaphylaxis [2]. Since the early 2000s, their prevalence has steadily increased [3], likely driven by both genetic and environmental factors—particularly in the first 1000 days of life, when microbial exposures, birth mode, feeding practices, and early antibiotic use can influence immune tolerance [4,5,6]. Current management relies on strict allergen avoidance and emergency treatment, which do not alter the natural course of the disease and impose major limitations on daily life [7]. The diagnostic process—often involving skin prick testing, specific IgE assays, and oral food challenges—is complex and burdensome for families [3,4,5]. Quality of life is frequently impaired [8,9,10], especially in children with multiple FAs, and precautionary food labeling inconsistencies across countries further increase uncertainty and anxiety [10,11,12,13]. In this landscape, omalizumab (OMA), a monoclonal anti-IgE antibody approved for allergic asthma and chronic urticaria, has emerged as a promising treatment for IgE-mediated FAs. By binding to circulating IgE and preventing its interaction with high-affinity FcεRI receptors on mast cells and basophils, OMA reduces allergic reactivity [14,15,16]. Clinical trials and real-world studies have demonstrated its efficacy in children both as standalone therapy and as an adjunct to oral immunotherapy (OIT), improving allergen tolerance and reducing adverse events during desensitization [15,16,17,18,19]. Despite growing interest, an up-to-date, pediatric-focused narrative review is currently lacking. No synthesis has comprehensively addressed OMA’s safety, efficacy, optimal dosing, integration with OIT, or impact on quality of life in children [20,21,22,23,24]. This review aims to fill that gap by providing a clinically oriented synthesis of current evidence. While not a systematic review, it follows a defined literature search strategy and explicit inclusion criteria to ensure transparency. Our goal is to support pediatricians, allergists, and other professionals in making evidence-based decisions regarding OMA use in children with food allergies. OMA’s mechanism of action allows not only for symptom control but also the possibility of reshaping clinical practice. By reducing the frequency and severity of reactions, it opens a “window of intervention” [14], enabling a proactive approach to treatment and improving psychological well-being. This represents a shift from reactive management (e.g., use of epinephrine after exposure) to anticipatory care, with potential long-term benefits for children and their families (Figure 1).

## 2. Materials and Methods

**Figure 2:**
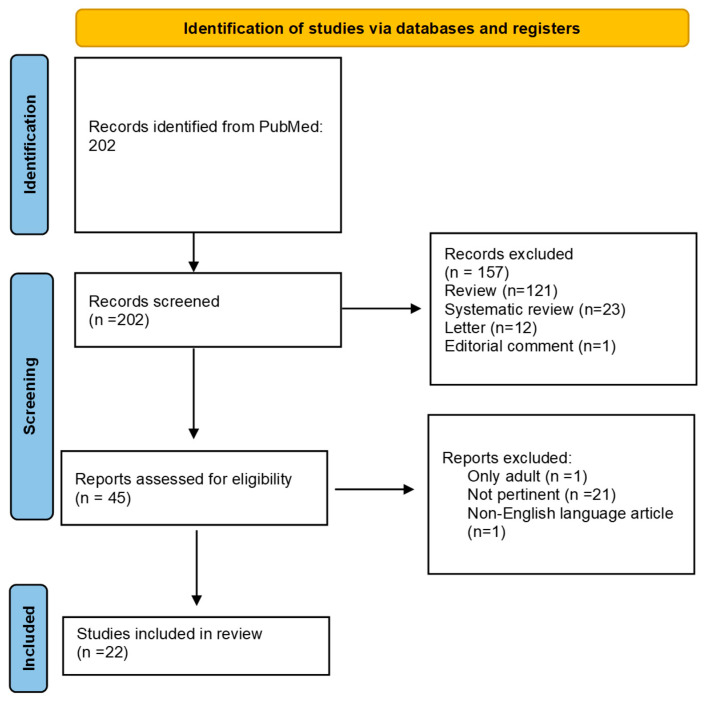
PRISMA Flowchart of Study Selection for Omalizumab in Pediatric Food Allergies.

A narrative literature review was conducted using a systematic search strategy to identify relevant studies on the use of OMA for FAs in children. The PubMed database was searched using the keywords “omalizumab”, “food allergy”, and “children”. To refine the search, specific filters were applied, including: case reports, clinical study, clinical trial, clinical trial protocol, controlled clinical trial, and observational study. The search was carried out between December 2024 and January 2025, without applying any time restrictions on the publication date of the articles. Although this review does not follow a systematic review methodology in the strictest sense (i.e., no meta-analysis was performed), we followed a systematic approach to article identification and selection to ensure comprehensiveness and transparency. The PRISMA flowchart (Figure 2) illustrates the study selection process, which summarizes the number of records identified, screened, excluded, and included. Three authors (A.K., P.P., and G.D.) independently reviewed the articles. Discrepancies in study inclusion were resolved through discussion and, if needed, in consultation with a fourth author (C.I.). Studies were included if they investigated the use of OMA in the context of FA and provided original clinical data involving pediatric populations or mixed-age groups (i.e., studies including both children and adults). All study designs were considered eligible, provided they reported relevant clinical outcomes related to food allergy management.

Exclusion criteria included: studies focusing exclusively on adult populations (“adult only”), studies not providing original clinical data (e.g., reviews, editorials), and studies considered not pertinent to the topic of FA (e.g., those dealing primarily with eosinophilic esophagitis or other non-IgE-mediated conditions).

## 3. Results

We included a total of 22 studies in our review, comprising 2 case reports [25,26], 5 case series or observational studies [27,28,29,30,31], and 15 interventional trials, including randomized controlled trials (RCTs), prospective observational designs, and cohort studies. These studies spanned a broad age range, from children as young as 1 year to adults up to 75 years old [18,32]. Most studies, however, primarily focused on pediatric and adolescent populations between 4 and 18 years [27,33,34,35]. In terms of intervention strategies, OMA monotherapy was evaluated in five studies [18,25,26,31,35]. These demonstrated consistent improvements in allergen tolerance, especially for common triggers such as peanuts, milk, and tree nuts. For example, Wood et al. (2024) [18] reported that 67% of participants treated with OMA tolerated ≥600 mg of peanut protein compared to just 7% in the placebo group. Mortz et al. [35] found statistically significant increases in allergen challenge thresholds, with all treated participants improving by at least two challenge steps (*p* = 0.003). A larger proportion of studies, 12 in total, investigated OMA in combination with oral immunotherapy (OIT) [27,28,33,34,36,37,38,39,40]. These trials showed that OMA coadministration not only enhanced desensitization success but also significantly improved safety profiles. For instance, in the study by Andorf et al. [33], 83% of participants in the OMA + OIT group tolerated ≥2 g of protein for two or more allergens, compared to 33% in the placebo group (*p* = 0.004). Similarly, MacGinnitie et al. [37] reported that 79% of the OMA group tolerated 2000 mg of peanut protein, and 75.9% succeeded at 4000 mg—contrasting starkly with 12.5% in the control group. Regarding the targeted allergens, peanuts were the most frequently studied food allergen, appearing in at least eight studies [18,32,33,35,37,39,41,42]. In these, participants commonly reached tolerance levels of 2000–4000 mg peanut protein following OMA treatment. Cow’s milk was the second most studied allergen, featuring in seven studies [27,28,36,38,39,40]. Other common allergens included tree nuts (e.g., cashew, hazelnut, walnut) [33,34,35], egg [34,35,40], and wheat [18,34]. Less frequent allergens included shellfish, fruits, and vegetables [26,30,43]. Results consistently indicated that OMA significantly enhanced food allergen tolerance, either by increasing challenge thresholds or improving the safety and success of OIT. Notably, Schneider et al. [42] found that 92% of participants reached a maintenance dose of 4000 mg peanut flour per day, with 85% tolerating 8000 mg after OMA discontinuation. Improvements extended beyond allergen tolerance: two studies also reported secondary benefits in asthma control [29,31], as measured by decreased inhaled corticosteroid use and improved quality of life scores. Dosage regimens for OMA varied across studies but were generally tailored based on body weight and total serum IgE levels. Dosing intervals typically ranged from every 2 to 4 weeks. Maximum doses varied, with most studies capping at 600–750 mg biweekly [38,40,41], while some adopted individualized dosing protocols based on immunologic markers such as CD-sens, measuring basophil allergen threshold sensitivity [25,44]. In the Azzano et al. [34] cohort, total IgE levels did not significantly influence initial escalation outcomes, but patients with a high specific/total IgE ratio had an elevated risk of adverse reactions during OMA tapering. Importantly, beyond allergen desensitization, several studies reported improvements in quality of life (QoL). For instance, Badina et al. [28] found that all participants showed improved scores on the Food Allergy Quality of Life Questionnaire (FAQLQ) by the end of the study. Similarly, Steiss et al. [31] documented improved QoL outcomes in terms of reduced school absenteeism, lowered reliance on emergency medications, and decreased asthma exacerbations. However, not all studies found improvements in QoL during the intervention period. Wood et al. [18] and Mortz et al. [35] reported no significant QoL changes during the blinded phases of their trials. Adverse events (AEs) were generally mild to moderate across studies, with OMA displaying a favorable safety profile. The most commonly reported AEs included injection site reactions, mild respiratory infections, or gastrointestinal symptoms. Serious adverse events were rare. For example, in Wood et al. [18], one serious event involving elevated liver enzymes occurred in a 1-year-old participant, though no severe events were reported in the open-label phase. Mortz et al. (2024) [35] recorded only mild AEs like viral infections and pneumonia, while Andorf et al. [33] reported a significantly lower rate of AEs in the OMA group compared to placebo (27% vs. 68%, *p* = 0.008). Nonetheless, caution is warranted, as some studies highlighted more severe reactions. Crisafulli et al. [27] noted that three participants experienced anaphylaxis, leading to discontinuation of therapy. In the trial by MacGinnitie et al., suspected cases of eosinophilic esophagitis were reported and resolved upon stopping peanut intake [37]. These findings underline the importance of close monitoring and individualized dosing strategies. OMA dosage regimens varied across studies but were generally tailored to body weight and total serum IgE levels. Dosing intervals ranged from every 2 to 4 weeks, with most studies capping maximum doses at 600–750 mg biweekly [38,40,41]. Some studies also explored individualized approaches, such as CD-sens-guided dosing based on basophil allergen threshold sensitivity [25,44], which appeared to reduce the risk of systemic reactions and optimize treatment efficacy. Azzano et al. found that, while total IgE levels did not predict initial escalation success, a high specific/total IgE ratio correlated with an increased risk of adverse reactions during OMA tapering [34].

Taken together, these findings support the role of OMA as both a monotherapy and adjunct to OIT, with demonstrable benefits in increasing food allergen thresholds and, in many cases, improving safety, comorbidity (asthma) control, and quality of life (Table 1).

## 4. Discussion

Our review, synthesizing findings from 22 clinical studies including randomized controlled trials and observational data, confirms that OMA, whether used as monotherapy or adjunctively with OIT, consistently improves food allergen tolerance and enhances the safety profile of desensitization protocols in children and adolescents [18,26,29,30,34,35,41,42,43]. These results underscore the role of OMA as a promising therapeutic option in the management of IgE-mediated food allergies, particularly in pediatric patients. While its efficacy in increasing allergen tolerance and reducing the severity of allergic reactions is clearly supported by the studies analyzed herein and the broader literature, a comprehensive evaluation of its overall impact is needed.

### 4.1. Efficacy of OMA in Food Allergy Management

OMA’s mechanism of action, binding to free IgE and preventing its interaction with FcεRI receptors, plays a crucial role in modulating the allergic response. By reducing mast cell and basophil activation, OMA decreases the release of inflammatory mediators responsible for allergic symptoms, thereby increasing the threshold for clinical reactivity to food allergens. The studies synthesized in this review consistently demonstrated this effect in pediatric populations, showing enhanced tolerance to common allergens such as peanuts, milk, eggs, and tree nuts [29,41]. In particular, OMA has shown efficacy in enabling patients to tolerate doses of peanut protein up to 4000 mg, representing a substantial improvement in allergen tolerance and safety during accidental exposures [18,29,37]. Furthermore, OMA has been shown to facilitate the success of OIT by reducing adverse reactions during treatment escalation phases. Studies incorporating OMA with multifood OIT indicate that a greater percentage of participants achieved desensitization to multiple allergens compared to OIT alone [33]. These findings highlight OMA’s potential to mitigate the risk of systemic allergic reactions, allowing for safer and more effective immunotherapy. Although the primary focus of our review is the pediatric population, it is important to note that several included studies also encompassed adolescents and adults and OMA’s efficacy, particularly for peanut allergy, has also been evaluated in adult cohorts [18,28,31,32,37,38,40,42,43,44]. This suggests potentially similar mechanisms of action and benefits, although specific protocols and outcomes may vary across different age groups. Our analysis, however, concentrates on pediatric data, where much of the recent research is focused and where the impact on quality of life and family dynamics is particularly significant.

### 4.2. Impact on Quality of Life

Living with food allergies imposes a significant psychological and emotional burden on patients and their families [8]. Fear of accidental exposure and anaphylaxis often leads to social restrictions and heightened anxiety. The role of psychological impact has gained great interest in recent years and has been evaluated through validated questionnaires [10,45,46]. The introduction of OMA into food allergy management offers a proactive approach that not only enhances safety but also alleviates the stress associated with food avoidance. Consistent with the broader literature, several observational studies included in our analysis specifically documented improvements in patient-reported outcomes, including reduced anxiety, increased dietary diversity, and improved overall quality of life [20,29,30,47]. Parents of children undergoing OMA therapy express greater confidence in their child’s ability to navigate daily activities without constant fear of severe allergic reactions. By reducing the psychological burden, OMA contributes to a more holistic approach to allergy management.

### 4.3. Economic Considerations and Patient Selection

One major limitation to consider is the economic impact of OMA treatment. As a high-cost biologic therapy, its widespread use in all patients with food allergies may not be feasible [22]. For this reason, OMA should be reserved for children with severe, multiple food allergies to primary food allergens, validated through recent scoring systems, who have experienced life-threatening reactions requiring emergency medical intervention, including the use of epinephrine and multiple emergency room visits [48,49]. Additionally, its use should be prioritized for patients with significantly impaired quality of life due to their dietary restrictions and fear of accidental exposure.

### 4.4. Clarifying the Role of OMA in Food Allergy Management

Another important consideration is the potential misinterpretation of OMA’s role in food allergy treatment. There is ongoing discussion regarding whether OMA enables liberalization of the diet to include the allergenic food. Currently, emerging evidence suggests that OMA should primarily be used as a preventive strategy to reduce the severity of allergic reactions following accidental ingestion rather than as a means to reintroduce the offending food into the diet without medical supervision [50,51]. Further studies are needed to clarify the extent to which OMA can support long-term tolerance versus its role as a protective measure against inadvertent exposure.

### 4.5. Regulatory Approval and Future Perspectives

Currently, OMA is approved for use in food allergy management only in the United States, following FDA approval. This regulatory milestone marks a significant advancement in the treatment of IgE-mediated food allergies [52]. However, in Europe, OMA is not yet approved for this indication, highlighting the need for further clinical trials and regulatory evaluations. We anticipate that additional studies conducted within the European context will provide the necessary evidence to support an approval by the European Medicines Agency (EMA), expanding access to this promising therapy for pediatric patients across different healthcare systems.

### 4.6. Considerations for Patients with High IgE Levels

A significant challenge in the use of OMA for food allergy management is the exclusion of patients with extremely high total IgE levels. In the United States, OMA is approved for IgE levels up to 700 IU/mL, while in Europe, the EMA has set a higher limit of 1500 IU/mL [53]. These limits are based on clinical trial data indicating that the current dosing tables for allergic asthma are based on weight and serum total IgE (t-IgE) levels prior to treatment. One of the main concerns with omalizumab is that, by reducing the levels of free circulating IgE, the drug may lead to an increased formation of immune complexes [16,54]. However, many patients with multiple food allergies exceed these thresholds, limiting their access to this potentially beneficial therapy [55]. Future studies should evaluate the safety and efficacy of OMA in patients with very high IgE levels to determine whether adjustments in dosing or alternative strategies could expand its applicability to this subset of patients [16,55].

### 4.7. Comparison with Other Therapeutic Strategies

Current food allergy treatments primarily focus on strict allergen avoidance and emergency management of anaphylaxis using epinephrine. While OIT offers a disease-modifying approach by gradually desensitizing patients to allergens, it carries a notable risk of adverse reactions, including gastrointestinal symptoms, systemic allergic responses, and eosinophilic esophagitis [17,27,56,57]. OMA has emerged as a complementary strategy that enhances the safety and efficacy of OIT, addressing key limitations of conventional immunotherapy. Unlike standalone OIT, OMA significantly reduces the incidence of dose-limiting reactions, allowing for more rapid and tolerable desensitization. Additionally, its potential as a long-term maintenance therapy for food allergy remains an area of interest, as preliminary evidence suggests that sustained OMA therapy may prolong the period of desensitization and reduce the likelihood of relapse upon discontinuation [51].

### 4.8. Limitations and Future Directions

Despite the promising outcomes observed in clinical studies, several limitations must be acknowledged. One major limitation is the variability in dosing regimens across different studies. OMA dosing is typically determined based on body weight and baseline IgE levels, but there is no standardized protocol for its use in food allergy treatment. This heterogeneity makes it challenging to establish uniform recommendations for clinical practice. Moreover, most studies evaluating OMA’s efficacy in food allergy treatment have relatively short follow-up periods. While initial desensitization and increased allergen thresholds are well-documented, the long-term sustainability of these effects remains unclear. Questions regarding the duration of OMA therapy necessary to maintain tolerance and the potential for permanent immune modification require further investigation. Additionally, while OMA reduces allergic reactions, it does not completely eliminate the risk of anaphylaxis. Patients undergoing treatment must still adhere to dietary precautions and carry epinephrine autoinjectors. The need for continued vigilance underscores the importance of combining OMA with comprehensive patient education and emergency preparedness. Finally, the literature search was conducted using only one database, PubMed, which may have limited the comprehensiveness of the findings. To optimize OMA’s role in food allergy management, future research should focus on refining treatment protocols, identifying biomarkers predictive of treatment response, and exploring its long-term immunological effects. Prospective studies should investigate whether early initiation of OMA in young children at high risk for food allergy could modify disease progression and potentially prevent the development of severe allergic phenotypes. Furthermore, health economic evaluations should assess the cost-effectiveness of OMA in the context of food allergy management. While its upfront costs are high, potential reductions in emergency room visits, hospitalizations, and caregiver burden may justify its inclusion as part of standard allergy care.

## 5. Conclusions

OMA represents a significant advancement in the treatment of IgE-mediated food allergies, offering a novel approach that not only increases allergen tolerance but also fundamentally improves patient outcomes. Findings from multiple clinical trials and observational studies demonstrate that omalizumab, whether administered as monotherapy or in combination with oral immunotherapy, significantly enhances allergen tolerance, reduces the risk of severe reactions, and improves the quality of life for both patients and their caregivers. Its efficacy in reducing adverse events and facilitating safer immunotherapy protocols underscores its potential as a valuable tool in allergy management. However, challenges related to long-term efficacy, cost, and treatment standardization remain. Future research should aim to address these gaps, paving the way for broader clinical application and enhanced care for individuals living with food allergies.

## Figures and Tables

**Table 1:** Clinical Evidence on Omalizumab for Food Allergy Management.

Reference	Type of Study	Number of Participants	Age (Range)	Intervention	OMA Dosage	Endpoints	Foods Involved	Duration	Results	Adverse Events
Wood et al., 2024 [18]	Double-blind, randomized, placebo-controlled trial	180 participants, of which 177 aged 1 to 17 years	1–55 years	OMA monotherapy	300 mg every 2 weeks or 225 mg every 4 weeks based on weight and IgE levels	**Primary endpoint**: To assess whether participants could tolerate at least 600 mg of peanut protein in a single dose without experiencing dose-limiting symptoms after 16–20 weeks of treatment. **Secondary endpoints**: To determine whether participants could tolerate at least 1000 mg of cashew, milk, and egg (each) without dose-limiting symptoms; to evaluate tolerance to increasing cumulative doses of one, two, or all three foods, up to a total of 6044 mg	Peanut, cashew, milk, egg, walnut, wheat, hazelnut	52 weeks	Increased threshold for peanut and other allergens; 67% achieved tolerance to ≥600 mg peanut protein vs. 7% placebo. At week 16, tolerance to a single dose of ≥1000 mg of food protein was achieved in the omalizumab group, by 41% for cashew (28/68 vs. 3%, 1/31), 67% for egg (34/51 vs. 0%, 0/20), and 66% for milk (27/41 vs. 10%, 2/21). No improvement in QoL was detected during the blinded phase	The safety profile was similar between the OMA and placebo groups, except for injection-site reactions, which were more common in the OMA group. One serious adverse event occurred in a 1-year-old participant who experienced elevated liver enzymes. No serious adverse events were reported during the open-label extension phase
Mortz et al., 2024 [35]	Randomized, double-blind, placebo-controlled	20	6–17 years	OMA monotherapy	Asthma-based dosing adjusted to total IgE and weight; doses every 2–4 weeks per protocol	**Primary endpoint**: To evaluate whether omalizumab increases the allergen threshold in food-allergic children after 3 months of treatment, assessed using a double-blind placebo-controlled food challenge (DBPCFC). **Secondary endpoints**: Changes in SPT size; Levels of specific IgE, specific IgG4 (s-IgG4), and total IgE (t-IgE); threshold changes at 6 months; QoL and atopic comorbidities	Peanut, hazelnut, cashew, walnut, egg	3–6 months	Significant increase in food allergy thresholds; all treated participants increased tolerance levels ≥2 challenge steps (*p* = 0.003) At 6 months, many children tolerated very high doses (up to 44,000 mg of food protein). Improvements were observed in SPT: significant reduction in wheal size (*p* < 0.02); s-IgG4: significantly increased (*p* < 0.006); s-IgE and t-IgE: increased as expected during anti-IgE therapy. No significant change in QoL	No significant differences in adverse events between the two groups. OMA group (n = 14): 5 children had mild adverse events within 3 months (viral infections, cystitis, constipation, pneumonia). From 3–6 months, 3 additional mild events occurred. No events occurred from 3–6 months
Andorf et al., (2018) [33]	Randomized, double-blind, placebo-controlled, phase 2	48	4–15 years	OMA combined with multifood OIT	Dosage based on weight and IgE levels, stopped by week 36	**Primary endpoint**: The efficacy of OMA combined with multifood OIT in passing DBPCFCs at week 36 from at least 2 of their offending foods. **Secondary endpoints**: Tolerance to higher doses; tolerance to more foods; successful completion of OIT with minimal symptoms; time to reach maintenance dosing: the speed at which participants were able to reach the 2 g maintenance dose per food	Cashew, walnut, hazelnut, almond, sesame, milk, egg, peanut, soy, wheat	36 weeks	83% of participants in the OMA group tolerated 2 g of protein for ≥ 2 foods vs. 33% in placebo (*p* = 0.004). All participants who passed the 2 g challenge (primary endpoint) also passed the 4 g challenge for at least 2 foods	No serious or severe adverse event were reported. AE incidence (weeks 8–16) was lower in the OMA group: 27% OMA vs. 68% (placebo) *p* = 0.008. No increase in AEs after OMA was discontinued at week 16
MacGinnitie et al. (2017) [37]	Randomized, double-blind, placebo-controlled, multicenter trial	37	7–19 years	OMA treatment followed by OIT for peanut allergy	Based on weight and IgE levels	**Primary endpoint**: Tolerance to peanut protein six weeks after discontinuation of OMA or placebo. **Secondary endpoint**: Tolerance to a greater dose of peanut protein during an oral food challenge 12 weeks after stopping treatment	Peanut	32 weeks	- 79% of OMA group tolerated 2000 mg of peanut protein vs. 12.5% of placebo group. - 75.9% of OMA group passed the 4000 mg challenge vs. 12.5% of placebo group	Adverse events were mostly mild and manageable, with allergic reactions occurring after 7.8% of OIT doses in the OMA group versus 16.8% in placebo. Moderate and severe reactions were more common in the placebo group, though not statistically significant. Three cases of suspected eosinophilic esophagitis resolved after stopping peanut intake
Brandström et al. (2017) [44]	Open-label, phase 2 study	23	12–19 years	Individually dosed OMA based on CD-sens monitoring	Adjusted based on IgE and weight. Final dose: up to 600 mg every 2 weeks	**Primary endpoint**: The suppression of peanut allergy symptoms, measured by a negative or favorable CD-sens test (basophil allergen threshold sensitivity) and the ability to undergo an open peanut challenge without severe allergic reactions. **Secondary endpoints**: The peanut protein dose tolerated during the challenge; the reduction in symptom severity and the dose adjustment strategy guided by CD-sens and IgE biomarkers, to optimize individual OMA dosing	Peanut	8–24 weeks	Significant reduction in allergen sensitivity: - Peanut challenge tolerance increased 50-fold on average. - All patients tolerated >840 mg of peanut protein	After OMA treatment, 78% of patients had no allergic symptoms during the peanut challenge, and the remaining 22% experienced only mild symptoms. No severe reactions occurred. The CD-sens-guided dosing strategy was both safe and effective, with no significant differences in adverse event rates between dosing groups
Crisafulli et al. (2019) [27]	Case series	8	7–15 years	OMA therapy combined with OIT	150–450 mg every 2–4 weeks based on IgE levels and weight	-	Cow’s milk	6–26 months	- Effective asthma control in all patients. - Two achieved milk tolerance; the others discontinued due to adverse reactions	Adverse events included local reactions at the site of injection and asthenia fever, headache, and generalized malaise after drug administration. Anaphylaxis occurred in 3 patients, leading to therapy discontinuation. Other adverse effects included nausea, urticaria, rhinitis, and asthma
Badina et al. (2022) [28]	Single-center, prospective, observational	4	8–24 years	OMA treatment followed by OIT	Based on s-IgE and weight; maximum 600 mg as per asthma guidelines	**Primary endpoint**: Facilitation of milk reintroduction by OMA **Secondary endpoint**: Increase in tolerated milk protein dose after 8 weeks of OMA; changes in immunological markers; improvement in asthma control	Cow’s milk	12 months	Improved tolerance to cow’s milk protein, no severe reactions during OIT, increased IgG4 levels, improved quality of life. Thresholds maintained post-OMA. Food Allergy Quality of Life Questionnaire (FAQLQ) scores improved in all four patients by the end of the study	No adverse effects related to omalizumab were recorded. One patient chose to reduce their milk intake post-OMA discontinuation due to anxiety
Sampson et al. (2011) [32]	Phase 2, randomized, double-blind, placebo-controlled	14 patients of which 8 were pediatric	6–75 years	OMA treatment followed by oral food challenge (OFC)	Minimum 0.016 mg/kg/IgE every 4 weeks, adjusted by IgE levels	**Primary endpoint**: Assess OMA’s efficacy in increasing peanut tolerance. **Secondary endpoints**: IgE reduction and safety	Peanut flour	24 weeks	44.4% of OMA-treated subjects tolerated ≥1000 mg of peanut flour after 24 weeks vs. 20% in placebo. Trend toward greater tolerance shift with OMA (*p* = 0.054). A significant decrease in free IgE was observed	OMA group: 76.5% experienced mild-to-moderate AEs; 2 cases of anaphylaxis occurred during pre-treatment oral food challenges (OFCs) Placebo group: 88.9% experienced mild-to-moderate AEs. No severe AEs reported during treatment
Nilsson et al. (2014) [25]	Case series	5	6–16 years	OMA treatment with CD-sens monitoring (basophil allergen threshold sensitivity)	Based on serum IgE and body weight; maximum 600 mg/2 weeks	**Primary endpoint**: Assess efficacy of OMA in achieving tolerance to cow’s milk in severe allergy. **Secondary endpoints**: Immunological changes and clinical improvements	Cow’s milk	16 weeks of OMA, continued for several years at lowest effective dose (75 mg/4 weeks) to sustain tolerance	All the patients achieved negative CD-sens and tolerated milk in challenges. 1 patient required doubled OMA dose to reach negative CD-sens. Reduction in IgE antibodies to milk proteins. CD-sens values dropped to 0 in all patients post-treatment. Asthma/rhino-conjunctivitis/eczema symptoms improved in most patients	No severe AEs attributed to OMA. The anaphylaxis risk was avoided during challenges due to CD-sens monitoring
Steiss et al. (2008) [31]	Observational study	9	8–17 years	OMA therapy for severe allergic asthma	Dosage based on body weight and IgE level: −150 to 750 mg every 2–4 weeks, depending on patient-specific factors (e.g., weight, IgE baseline)	**Primary endpoint**: Reduction of total IgE levels during OMA therapy in children/adolescents with severe allergic asthma. **Secondary endpoints**: Asthma exacerbations; rescue medication use and comorbid allergy management	Peanut, others not specifically mentioned	6 months up to 24 months	Significant decrease in total IgE after 6 months of therapy (*p* < 0.01) - Reduction in asthma exacerbations and need for rescue medications - Improved quality of life (reduced school absenteeism and decreased reliance on emergency medications). - Lowered inhaled glucocorticoid doses	No severe AEs reported. No systemic reactions to subcutaneous injections
Frischmeyer-Guerrerio et al. (2017) [38]	Randomized, placebo-controlled study	57 patients of which 40 aged 7–17 years old	7–35 years	OMA in combination with OIT for milk allergy	0.016 mg/kg/IgE IU (max dose ≤750 mg)	**Primary endpoint**: Investigate mechanistic effects of OMA combined with milk OIT and identify baseline biomarkers predictive of clinical benefit. **Secondary endpoints**: Safety and immunological changes	Cow’s milk (casein)	OMA administered for 16 months (combined with OIT from month 4–28). OIT continued for 24–32 months	OMA significantly suppressed milk-induced basophil CD63 expression at month 4 (before starting OIT), with *p* values of 0.0074 and 0.0135 for 0.1 and 10 mg/mL milk, respectively. Compared to placebo, CD63 expression was also significantly lower at various concentrations (*p* = 0.0040 to 0.0014). Histamine release was significantly increased in OMA-treated subjects upon stimulation with milk and anti-IgE (*p* values ranging from 0.0035 to 0.0302), suggesting enhanced basophil sensitivity in washed cell preparations	In the OMA group, reduced moderate/severe reactions vs. placebo (*p* < 0.01) were observed. No severe AEs attributed to OMA. In the placebo group higher rates of gastrointestinal and systemic symptoms during OIT were noted
Azzano et al. (2021) [34]	Cohort study	181	5.2–13.9	OMA pre-treatment (≥2 months) to OIT	Weight (and IgE)-based dosage	**Primary endpoint**: Identify determinants of OMA dose-related efficacy in OIT for food allergy. **Secondary endpoint**: Pharmacokinetics/pharmacodynamics	Up to 6 allergens simultaneously (e.g., peanuts, egg, milk, tree nuts)	OMA started ≥2 months pre-OIT. The total duration varied by patients	- OMA dosage per weight alone correlated strongly with progression in initial food escalation (IFE). - Total IgE levels did not affect IFE outcomes. - Free IgE levels and OMA-IgE complexes predicted IFE outcomes. - Patients with a high s-IgE/t-IgE ratio had an increased risk of reaction during OMA weaning. - Sustained food consumption was 90.3% after 2 years	118/181 (65%) had reactions; only 8 (4.4%) were moderate/severe. No severe AEs attributed to OMA
Takahashi et al. (2017) [36]	Randomized controlled trial	16	6–14 years	OIT combined with OMA (OMA-OIT)	Dose calculated based on total IgE and body weight. Typically administered every 2–4 weeks for 24 weeks	**Primary endpoint**: Induction of desensitization to cow’s milk at 32 weeks after study entry. **Secondary endpoints**: Change in successfully consumed dose; immunologic responses and safety	Cow’s milk	32 weeks (24 weeks of OMA + OIT, followed by 8 weeks of OIT alone)	All 10 children in OMA-OIT group desensitized; none in control group desensitized. Wheal size reduced in OMA-OIT group. Significant increase in tolerance for CM protein (median 2080 mg vs. 0 mg in controls; *p* < 0.001) and fresh CM (200 mL vs. 0 mL; *p* = 0.006)	No severe AEs reported
Wood et al. (2016) [40]	Randomized, double-blind, placebo-controlled trial	57	7–32 years. Not specified how many patients were pediatric	OMA combined with milk oral immunotherapy (MOIT)	Based on weight and IgE level; max 750 mg every 2–4 weeks	**Primary endpoint**: Passing a 10 g milk protein oral food challenge (OFC) after 8 weeks off OIT. **Secondary endpoints**: Desensitization at month 28 and safety/adverse events	Cow’s milk	32 months	Significant safety improvements: fewer dose-related reactions. No significant difference in desensitization or sustained unresponsiveness (SU) between the two groups	OMA group had fewer and milder reactions. No severe reactions requiring discontinuation. Epinephrine use: 2 doses (OMA) vs. 18 doses (placebo)
Nadeau et al. (2011) [39]	Pilot phase 1 study	11	7–17 years	OMA combined with OIT	Dosed every 2–4 weeks; based on weight and IgE level (up to 300 mg)	**Primary endpoint**: Desensitization to 2000 mg/day of milk protein within 7–11 weeks of starting OIT. **Secondary endpoints**: Safety and long-term tolerability	Cow’s milk	52 weeks	9 of 11 participants achieved desensitization to 2000 mg/day in 7–11 weeks. All 9 patients could tolerate ≥8000 mg/day after discontinuation of OMA	Low reaction rate (1.6%), mostly mild. Epinephrine needed in 3/11 subjects
Schneider et al. (2013) [42]	Pilot study	13	7–16 years	Oral peanut desensitization with OMA pre-treatment	Administered every 2–4 weeks based on European guidelines. Doses ranged from 150–600 mg depending on IgE levels and weight	**Primary endpoint**: First-day desensitization to 500 mg peanut flour. **Secondary endpoints**: Achieving maintenance dose (4000 mg/day); long-term tolerance (8000 mg challenge at Week 32) and safety	Peanut	48–156 weeks of OMA treatment continued for median of 4 years	92% reached a maintenance dose of 4000 mg peanut flour/day. Tolerance to 8000 mg (approx. 20 peanuts) achieved in 85% of participants after OMA discontinuation	Reaction rate: 2% of doses (72 reactions total): mild 1.8% (no treatment needed), moderate 0.2% (antihistamines used), severe 0.06% (epinephrine used in 2 cases)
Brandström et al. (2019) [41]	Phase 2 study	23, 13/23 (57%) were ≤17 years old	12–19 years	Peanut oral immunotherapy (pOIT) with OMA pre-treatment	Individualized, adjusted every 2–4 weeks, based on IgE levels and clinical response	**Primary endpoint**: Tolerating pOIT for 12 weeks after stopping OMA, followed by a negative open peanut challenge (2800 mg). **Secondary endpoint**: Safety, immunological markers, clinical outcomes	Peanut	8-week up-dosing phase, with an individualized tapering schedule afterward	48% achieved the primary endpoint of tolerating 2800 mg peanut protein 12 weeks after stopping OMA. Adverse reactions increased when OMA was reduced or stopped	Systemic reactions: 43 reactions in 16,095 doses (0.3%): mild: 20 (0.1%). Moderate: 22 (0.1%). Severe: 1 (0.006%). Common symptoms: oropharyngeal and abdominal. Adrenaline use: 8/23 (35%) required adrenaline
Rafi et al. (2010) [43]	Prospective pilot study	22 patients, of which 5 were pediatric	14–66 years	Treatment of IgE-mediated food allergies in asthma patients using OMA	150–300 mg every 2–4 weeks	**Primary endpoint**: Reduction or elimination of allergic symptoms upon accidental or intentional reexposure to sensitized foods. **Secondary endpoints**: Symptom-specific improvements and safety	Fish, shellfish, peanut, tree nuts, egg, soybean, wheat	At least 1 year	100% of patients (22/22) showed improvement by the 6th dose. Food-induced asthma exacerbations improved in 13/22 (59%) patients. Atopic dermatitis: improved in 8/22 (36%) patients. Rhinosinusitis: Improved in 6/22 (27%) patients. Urticaria: improved in 3/22 (14%) patients. Angioedema/anaphylaxis: improved in 9/22 (41%) patients	Common AEs: injection site reactions, viral infections, upper respiratory infections, sinusitis, headaches. Serious AEs (SAEs): anaphylaxis risk (required 2 h post-injection monitoring)
Arasi et al. (2024) [29]	Observational study	69	6–18 years	OMA therapy for food allergies in asthmatic children	Dosage adjusted per baseline IgE and weight, capped at t-IgE of 1500 IU/L before June 2021, later ≤2500 IU/L	**Primary endpoint**: Increase in threshold of reactivity (NOAEL) to food allergens after 4 months of OMA treatment, measured via oral food challenges (OFCs). **Secondary endpoints**: Proportion of negative OFCs at T1 (tolerance without reaction). NOAEL increase at T2 and T3 in patients who failed T1 challenges. Reduction in anaphylactic episodes (pre-treatment vs. post-treatment). Reduction in non-anaphylactic food reactions	Peanut, tree nuts, fish, egg, milk, wheat	1 year	Increased tolerance to foods: 66.4% of foods tolerated at T1; quality of life (FA-QoL): children ≤12 years: improved from 4.63 ± 0.74 to 2.02 ± 1.13; adolescents: improved from 4.68 ± 0.92 to 1.90 ± 1.50. Significant improvements noted across physical, emotional, and social domains	None reported related to omalizumab administration
Sakamoto et al. (2023) [26]	Case report	1	12 years	Sublingual immunotherapy (SLIT) combined with OMA	600 mg/month	Improvement/resolution of intractable lip edema caused by pollen–food allergy syndrome (PFAS)	Oranges, apples, peaches, tomatoes, strawberries, tomato sauce	4 years	Marked improvement in lip edema and nasal symptoms; no relapse of lip edema for ~2 years	No adverse reactions reported from OMA during the study period
Crespo et al. (2021) [30]	Observational study	5	4–8 years	OMA treatment with OFC	150–300 mg every 4 weeks; adjusted per patient	**Primary endpoint**: Effectiveness of OMA in enabling tolerance to previously allergenic foods in children with multiple food allergies (MFAs), evaluated through oral food challenges (OFCs). **Secondary endpoints**: Changes in total and specific IgE levels. Introduction of foods previously excluded due to anaphylaxis. Ability to reduce OMA dose or extend administration interval	Various foods including nuts, fish, vegetables, fruits, and cereals	2.3 to 6.2 years	Significant increase in food tolerance, reduction in allergic reactions, improved quality of life	No local or systemic adverse events related to OMA reported
